# Emission of Volatile Organic Compounds in Crumb Rubber Modified Bitumen and Its Inhibition by Using Montmorillonite Nanoclay

**DOI:** 10.3390/polym15061513

**Published:** 2023-03-18

**Authors:** Gang Liu, Shuaiyin Fang, Yong Wang, Jinjun Liu, Yangshi Liang, Tingwei Cao, Quantao Liu

**Affiliations:** 1 State Key Laboratory of Silicate Materials for Architectures, Wuhan University of Technology, Wuhan 430070, China; 2 College of Water and Architectural Engineering, Shihezi University, Shihezi 832003, China; 3 School of Materials Science and Engineering, Wuhan University of Technology, Wuhan 430070, China

**Keywords:** VOCs, crumb rubber, bitumen, organic montmorillonite, inhibition

## Abstract

Bitumen emits a large amount of volatile organic compounds (VOCs) during the production and construction of asphalt mixture, which can cause both environmental hazards and health risks. In this study, a setup was designed to collect the VOCs released by base and crumb rubber-modified bitumen (CRMB) binders and their composition was characterized by thermal desorption-gas chromatography-mass spectrometry (TD-GC-MS). Next, organic montmorillonite (Mt) nanoclay was added into CRMB binder and its inhibiting effect on the VOCs emission of the binder was investigated. Finally, the VOCs emission models for the CRMB and Mt-modified CRMB (Mt-CRMB) binders were established according to reasonable assumptions. The results indicated that the VOCs emission of CRMB binder was 3.2 times larger than that of the base binder. Due to its intercalated structure, the nanoclay can reduce the VOCs emission of CRMB binder by 30.6%. Especially, its inhibition effects on alkanes, olefins, and aromatic hydrocarbons were more significant. After finite element verification, the established model based on the Fick’s second law can describe the emission behavior of CRMB and Mt-CRMB binders well. Overall, the Mt nanoclay can be used as an effective modifier to inhibit the VOCs emission of CRMB binder.

## 1. Introduction

Every year, over 1.6 billion new tires and around 1 billion waste tires are generated [1]. Approximately 18 million tons of waste tires in China are produced yearly, but their recycling rate is less than 40% [2]. The accumulation of a large number of waste tires occupies land resources, and it has the risk of auto-ignition, which can produce a large amount of harmful smoke [3]. At present, a broad application of the waste tires is to produce crumb rubber (CR) powder to modify bitumen binder. Many studies have shown that asphalt mixture with crumb rubber-modified bitumen (CRMB) binder can effectively improve the resistance to the high-temperature deformation, fatigue performance, and rheological performance [4,5,6,7].

Bitumen can emit large amounts of volatile organic compounds (VOCs) during the production of asphalt mixture and the construction of asphalt pavement [8]. The CRMB mixture needs a higher production temperature, and its VOCs emission is larger than the traditional hot-mix asphalt [9]. Bitumen is composed of saturate, aromatic, resin, and asphaltene. Among them, aromatic is the most harmful and contains benzene, naphthalene, anthracene, phenanthrene, pyridine, acridine, carbazole, phenol, and other volatile substances [10]. With long-term exposure to such an environment, the harmful substances will enter the human body through skin contact and the respiratory tract, seriously harming the health of humans [11,12]. The influence of bitumen fumes, including VOCs, is attracting more and more attention. The International Agency for Research on Cancer listed bitumen fumes as a suspected carcinogen in 1987 [13]. In 2015, China implemented new regulations (GB31570-2015), which set the emission standard of asphalt fumes at 20 mg/m^3^ [14,15]. Therefore, the emission problem of bitumen VOCs is one of the important factors limiting the further expansion of bitumen applications.

Lots of research has been carried out on how to inhibit the emission of bitumen VOCs. Temperature is an important factor affecting the VOCs emission of bitumen [16]. Compared with the traditional hot-mix asphalt (HMA), the warm-mix asphalt (WMA) technology can reduce the construction temperature by 20–40 °C, down to 100–140 °C [17]. The WMA containing the CR powder can effectively reduce polycyclic aromatic hydrocarbon emissions [18]. However, the warm-mix agents decreased the low-temperature cracking and moisture damage resistances of the asphalt mixture [19]. The nano-sized calcium carbonate and styrene butadiene styrene copolymer (SBS) can be used to absorb the VOCs emission of bitumen [20]. However, SBS polymer is susceptible to aging and degradation at high temperatures, which will reduce the inhibition effect of SBS on bitumen VOCs [21,22]. In addition, some inorganic materials such as zeolite, layered double hydroxides, and expanded graphite have been proven to be used as asphalt VOCs inhibitors, but the compatibility between the inorganic materials and asphalt is poor, tending to cause segregation problems at high temperatures [23,24,25].

Montmorillonite (Mt) is a widely available and inexpensive mineral. Its unit cell has a crystal structure comprising a 2:1 layered silicate, two silicon-oxygen tetrahedron layers, and a sandwiched layer of aluminum-oxygen octahedrons [26,27]. The surface of its silicate lamellar layer is negatively charged and is composed of accumulation by electrostatic interaction between layers [28]. Montmorillonite has unique one-dimensional layered nanostructures and cation exchange properties, which makes it possible to modify and expand the application field [29,30]. Modification by organic compounds can significantly improve its utilization rate. It has been reported that organic Mt can improve the anti-aging performance of bitumen, and the anti-aging performance was better than SBS-modified bitumen when the content exceeded 3% [31]. In addition, the physical and chemical stability of montmorillonite sheet can further enhance the flame-retardant performance of polymers [32]. Moreover, 5 wt.% Mt containing alkyl surfactants reduced VOCs emissions of base bitumen by more than 50% through interlayer restriction of volatilization of the light components of the bitumen [33]. Organic Mt with a particle size greater than 200 mesh size significantly reduced the types and quantities of SBS-modified bitumen VOCs [34]. The research above indicates that Mt can not only effectively improve the performance of bitumen, but also has great potential in inhibiting VOCs emission. However, using Mt to inhibit the VOCs emission of CRMB has not been reported.

This study aimed to characterize the VOCs emission of CRMB binder and evaluate the effect of Mt nanoclay on its inhibition. Firstly, a homemade setup was used to collect the VOCs of base and CRMB binders during the heating process, and their composition was characterized. Next, nanoclay was used to modify the CRMB binder, and its inhibition effect on the VOCs emission of the CRMB binder was evaluated. Finally, based on the Fick’s second law, the VOCs emission models for the CRMB and Mt-modified CRMB (Mt-CRMB) binders were established. 

## 2. Experimental

### 2.1. Materials

Base bitumen (IRPC-70) with a penetration grade of 70 was imported from Thailand and used in this study. The basic properties of the base bitumen are presented in Table 1.

One waste tire from a passenger car was chosen to produce the CR powder. Its preparation process was as follows: first, the electric grinder was adopted to grind the tread part of the waste tire to produce the CR powder; next, the magnet was used to remove iron impurities; then, the CR powder was sieved and collected with a typical particle size between 40 and 60 mesh number (i.e., 250–425 μm) to be used in this study; finally, it was washed by the water to remove other impurities (e.g., dust) and dried at 60 °C in the oven for 12 h.

The organic Mt produced by Zhejiang Fenghong Clay Chemical Co. Ltd. (Huzhou, China) was used to modify the CRMB binder, and distearylammonium chloride was used as the surfactant with the content of 9%. The physical properties of montmorillonite are presented in Table 2.

### 2.2. Modified Bitumen Preparation

During the preparation of the CRMB or Mt-CRMB binders, 250 g of base binder was melted and poured into a cylindrical container on the heating plate. Then, 45 g of CR powder (18% by weight) was added to the base binder, dispersing with a low-speed mixer (JJ-1, Shanghai Loikaw Instrument) at 200 rpm for 10 min, at a temperature of 150 °C. For the Mt-CRMB binder, 10 g of Mt (4% by weight) was simultaneously added with the CR powder into the base binder. Next, the temperature was increased to 180 °C, and the binder was sheared with a high-speed shearing mixer (ESR-500, Shanghai ELE Mechanical and Electrical Equipment, Shanghai, China) at 4500 ± 500 rpm for 1 h. All samples were cooled to room temperature and stored for further testing. More information about the bitumen binder modification process in this study can be found in the previous research [33].

### 2.3. Test Methods

#### 2.3.1. VOCs Collection Methods

**Figure 1:**
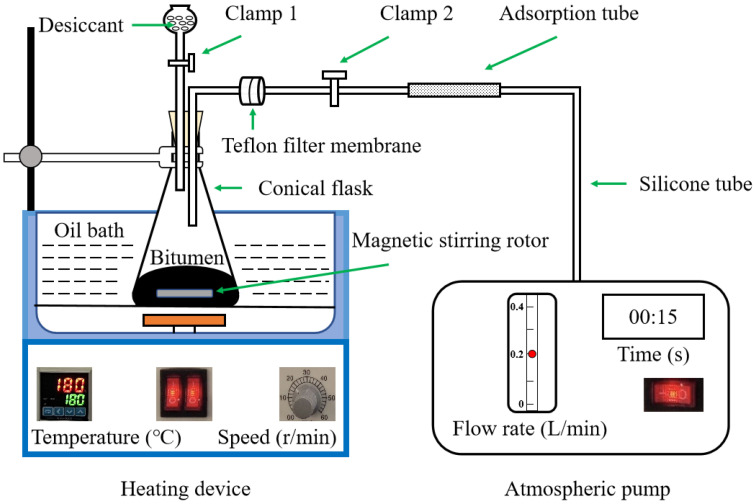
VOCs sample collection device.

The VOCs sample collection device is shown in Figure 1. Two VOCs sampling methods were used in this study. Method 1 was used to compare the VOCs characteristics of CR powder, base, and CRMB binders, by opening or closing the clamps, and Method 2 was not only used to characterize the VOCs emission of the binders at different heating times, but also could be used to calculate the VOCs emission rates in order to understand the entire VOCs emission behavior. The specific collection processes for these two methods are outlined below.

Method 1: First, 50 g of binder or the CR powder sample was put into the conical flask, and Clamps 1 and 2 were closed (see Figure 1). When the temperature of the heating device rose to 180 °C, the magnetic stirring switch was turned on and the temperature was held for 10 min. Then, the two clamps were opened, and the VOCs were adsorbed into the ATD (automatic tube dispenser) by using an atmospheric pump at a gas flow rate of 0.2 L/min for 15 s.

**Figure 2:**
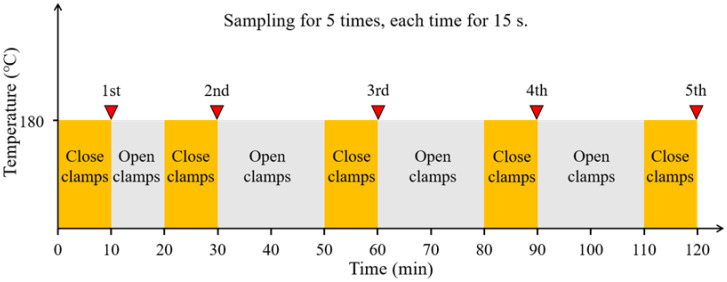
Operation process for Method 2 (sampling five times).

Method 2: The CRMB binder or Mt-CRMB binder with the weight of 50 g was poured into the conical flask (see Figure 1). As shown in Figure 2, the VOCs at 180 °C were collected at five sampling points (i.e., 10, 30, 60, 90, and 120 min) to simulate the VOCs release characteristics of the asphalt mixture during the process of mixing, transporting, and paving. The collection time for each point was 15 s, with a flow rate of 0.2 L/min using the pump. Before each collection, the clamps were closed for 10 min to preserve enough VOCs to be absorbed by the ATD.

#### 2.3.2. VOCs Characterization

Atomx P&T-Agilent 7890B-5977B thermal desorption-gas chromatography-mass spectrometry (TD-GC-MS, Agilent, Santa Clara, CA, USA) was used to characterize the VOCs emission of the binder. The gas temporarily adsorbed in the automatic tube dispenser (ATD, PerkinElmer, Waltham, MA, USA) was desorbed in the thermal desorption system and then transferred to the ultra-inert capillary column (30 m × 250 mm × 0.25 mm) using high-purity helium (≥99.999%) at a flow rate of 40 mL/min. The GC-MS system was kept at 30 °C for 3 min and then heated up to 200 °C at 11 °C/min for 3 min. Finally, it was kept at 280 °C for 5 min. The ion-source temperature was maintained at 230 °C, and the MS detector (Agilent, Santa Clara, CA, USA) was operated in full-scan electron ionization (EI) mode, where data over 35–400 m/z were acquired [35,36].

#### 2.3.3. X-ray Diffraction (XRD) Test

A D/Max-RB X-ray diffractometer (Rigaku, Tokyo, Japan) was used to analyze the structural changes of the organic montmorillonite in the CRMB binder, with the conditions: Cu Kα rays (λ = 1.54184 Å), the tube voltage of 40 kV, the tube current of 40 Ma, the step size of 0.02°, the scanning range of 1–10° (2θ), and the scanning rate of 5°/min.

## 3. Results and Discussion

### 3.1. VOCs Composition of Base and CRMB Binders

**Figure 3:**
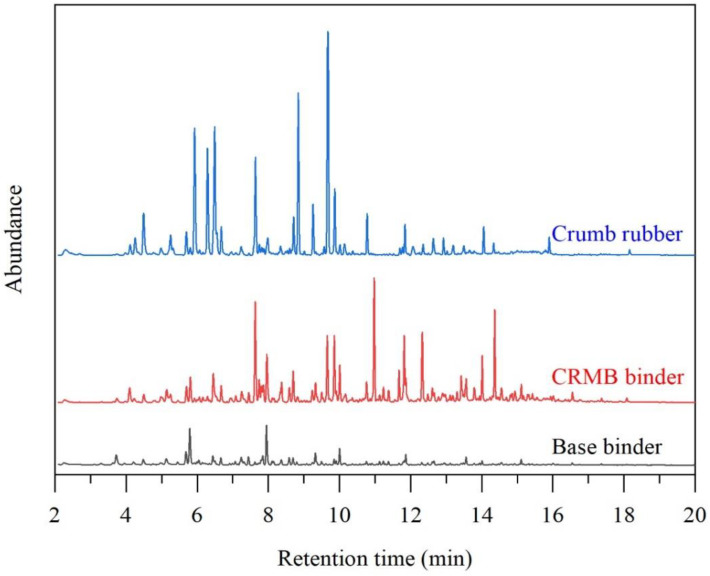
VOCs GC-MS chromatograms of CR powder, base, and CRMB binders.

Figure 3 shows the GC-MS chromatograms of VOCs emitted by the base binder, CRMB binder, and pure CR powder. Based on the NIST database, there were 123 organic compounds detected in the base binder VOCs. Commonly, the bitumen VOCs can be classified into alkanes (ALK), olefins (OLE), hydrocarbon derivatives (HYD), aromatic hydrocarbons (ARH), and aromatic hydrocarbon derivatives (ARHD). Among these compounds, there were 49 ALKs, 31 OLEs, 19 HYDs, 24 ARHs, and 0 ARHD. It was indicated that most compounds in the base binder VOCs belonged to the ALK and OLE.

For the CR powder, 116 VOCs were detected, containing 17 ALKs, 29 OLEs, 62 HYDs, 6 ARHs, and 2 ARHDs. It was indicated that the hydrocarbon derivatives dominated the VOCs of CR powder. Meanwhile, there were two aromatic hydrocarbon derivatives.

The GC-MS results indicated that there were 144 compounds in the CRMB binder VOCs. Among them, there were 42 ALKs, 43 OLEs, 28 HYDs, 29 ARHs, and 2 ARHDs. Compared with the base binder VOCs, all the species numbers increased, except in the ALE. Maybe some alkane compounds in the binder were absorbed by the rubber, and the ALE volatilization decreased. Two aromatic hydrocarbon derivatives were detected, possibly originating from the CR powder with the same VOCs.

In terms of ionic peak intensities, the CR power, base binder, and CRMB binder VOCs were significantly different. For the CR powder VOCs, the abundances of methyl acrolein, methyl vinyl ketone, benzene, 2-ethenyl-2-butenal, and methyl isobutyl ketone were larger. For the base binder VOCs, the abundances of n-hexane and heptane were larger. For the CRMB VOCs, the abundances of benzene, 4-ethenyl-cyclohexene, and D-limonene were larger.

**Figure 4:**
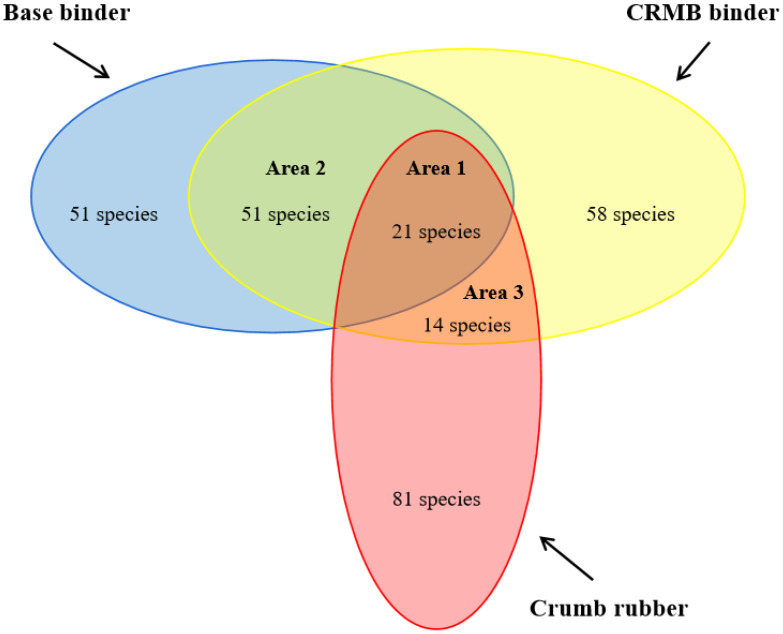
VOCs species overlap for crumb rubber, base, and CRMB binders.

Figure 4 shows the distribution of VOCs species for the CR powder, base, and CRMB binders. Three areas were defined: Area 1 meant the overlap of 21 VOCs among the CR powder, base, and CRMB binders, Area 2 meant 51 joint VOCs between the base and CRMB binders, and Area 3 represented 14 joint compounds between the CR powder and the CRMB binder. It was noted that 51 VOCs in the base binder and 83 VOCs in the CR powder were not detected in the CRMB binder. However, there were 58 new VOCs in the CRMB binder. 

**Figure 5:**
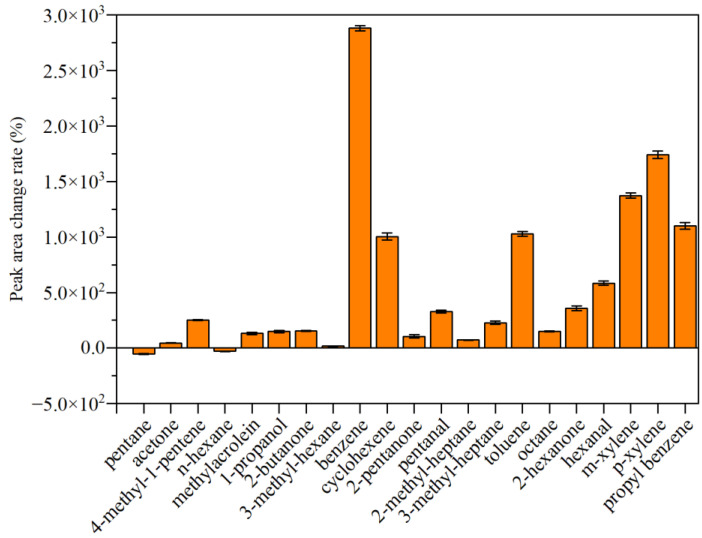
Peak area changes of 21 compounds in Area 1 based on the GC-MS chromatograms of the CRMB binder.

Figure 5 shows the peak area changes of 21 compounds in Area 1 based on the GC-MS chromatograms of the CRMB binder. It indicated that except for pentane and n-hexane, the peak areas of the other 19 compounds increased compared with the base binder. Among them, the peak areas of benzene, cyclohexene, toluene, m-xylene, p-xylene, and propyl benzene increased by 28 times, 10 times, 10 times, 14 times, 17 times, and 11 times, respectively. It can be seen that because the waste CR powder itself contains these compounds, adding the CR powder to the base binder will have a direct impact on the emissions of these compounds in VOCs.

The CR powder mainly introduced 14 VOCs in Area 3. Among them, ethanol, methyl isobutyl ketone, ethyl acetate, 1-pentanol, and D-limonene are often used as essential solvents in the rubber tire. The 4-ethenyl-cyclohexene and styrene are critical raw materials for synthesizing rubber. Benzothiazole was used as the rubber vulcanization accelerant, while 2-methyl-furan, heptanal, octanal, nonanal, (E)-6-dodecane, and 2-ethenyl-2-butenal were used as the intermediates in the rubber synthesis.

Based on the previous research [37,38,39], 23 normally used VOCs were selected as the fingerprint compounds for the CRMB binder (see Table 3). Among them, there were 12 species from Area 1, 5 from Area 2, and 6 from Area 3.

The external method was used to obtain the standard curve of the fingerprint compound. First, a standard solution was prepared by mixing 23 fingerprint compounds in the ethanol solvent. Each compound had 5, 10, 25, 50, and 100 ppm concentrations. For other non-fingerprint compounds, the standard curve of toluene was used [9]. After the GC-MS test, the standard curve describing the relationship between the peak area and mass of each compound was obtained as follows:

(1) $$m_{i}=\frac{S_{i}}{k_{i}}$$

where,$\;m_{i}$ is the mass of fingerprint compounds, ng, $S_{i}$ is the peak area of fingerprint compounds, and $k_{i}$ is the slope of the standard curve for fingerprint compounds, ng^−1^.

The total amount of bitumen VOCs emission was calculated as follows:

(2) $$m=\sum_{i=1}^{n}m_{i}+\frac{S_{t}-\sum S_{i}}{k_{tol}}$$

where, m is the total amount of bitumen VOCs emission, ng, n is the number of fingerprint compounds, $S_{t}$ is the total peak of all VOCs, and $k_{tol}$ is the slope of the standard curve measured by the standard toluene solution prepared in this study, ng^−1^.

**Figure 6:**
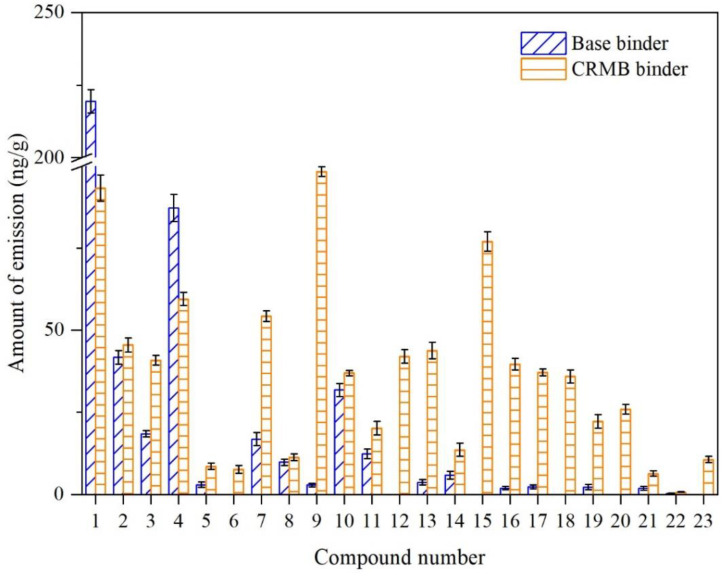
Emission comparison of each VOC fingerprint compound between the base and CRMB binders.

Figure 6 shows the VOCs emission amount of each fingerprint compound. The total base binder VOCs amount was 1306 ng/g. However, this amount for the CRMB binder VOCs was 4180 ng/g, three times that of the base binder. It was indicated that the addition of CR significantly increased the total emission of bitumen VOCs. Two lightweight compounds (No. 1 pentane and No. 4 n-hexane) in the CRMB binder VOCs had a lower emission amount, decreasing by 57.47% and 31.79%, respectively. The reason was that the CR powder absorbed part of the lightweight compounds in the base bitumen, thereby reducing their emissions. However, most fingerprint compounds had an increased emission. The emission amount of benzene, toluene, m-xylene, p-xylene, and mesitylene in the CRMB binder VOCs increased by 34, 11, 20, 15, and 9 times, respectively. It indicated that the CR powder significantly influenced the ARHs emission.

### 3.2. Inhibition Effect of Mt on the VOCs Emission of CRMB Binder

**Figure 7:**
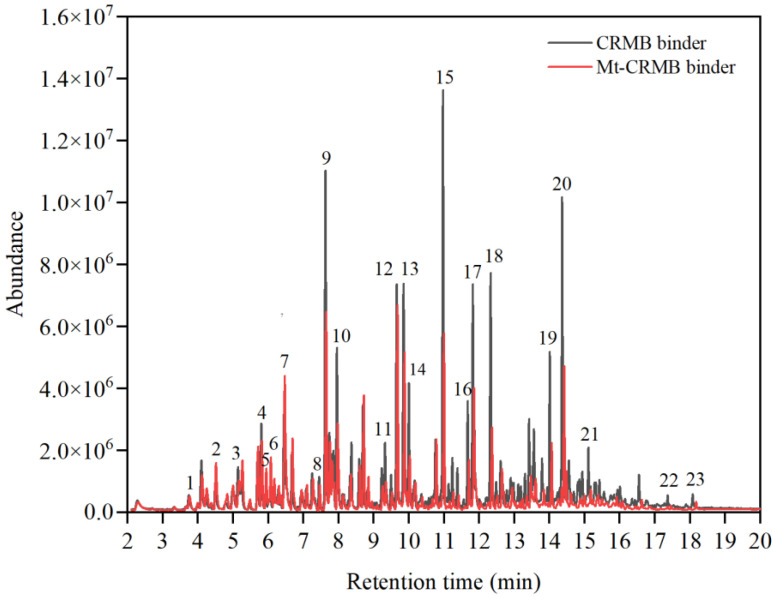
VOCs GC-MS chromatograms of CRMB and Mt-CRMB binders.

Figure 7 shows the VOCs GC-MS chromatograms of the CRMB and Mt-CRMB binders. The results indicated that Mt-CRMB VOCs had 108 compounds, 25% less than the CRMB VOCs, which had 144 compounds. It can be easily observed that most fingerprint compounds of the Mt-CRMB VOCs had a smaller peak than that of the CRMB VOCs. Calculated by Equation (2), the total emission of the Mt-CRMB binder was 2901 ng/g, 30.6% less than that of the CRMB binder.

**Figure 8:**
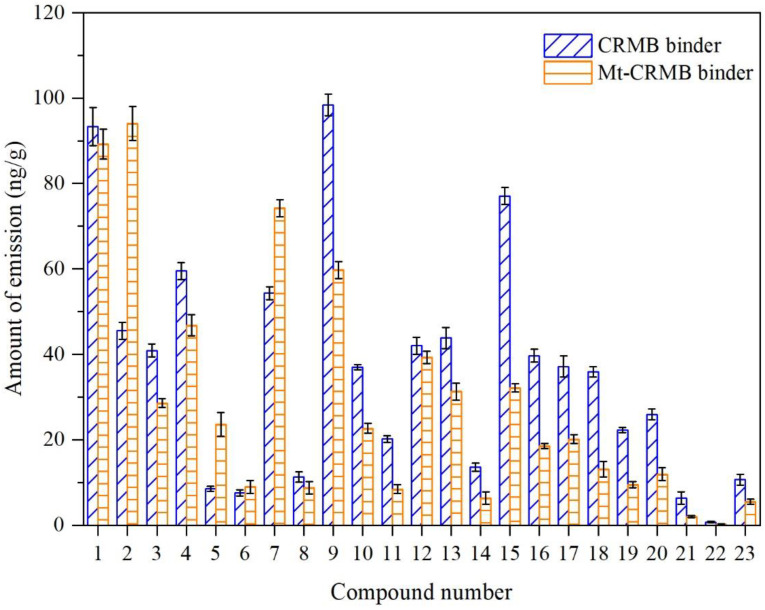
Emission amount comparison of each VOC fingerprint compound between CRMB and Mt-CRMB binders.

Figure 8 compares the emission amount of each VOC fingerprint compound between CRMB and Mt-CRMB binders. Due to the addition of Mt, 19 compounds had a decreased emission. Among them, the emission of compounds Nos. 3, 9, 10, 11, 14, 15, 16, 17, 18, 19, 20, 21, 22, and 23 was significantly reduced by more than 30%. These compounds belonged to the ALK, OLE, and ARH. However, the inhibition effect of Mt on the compounds of No. 2 (acetone), No. 5 (methacrolein), No. 6 (2-methyl-furan), and No. 7 (butanal) was not good.

**Figure 9:**
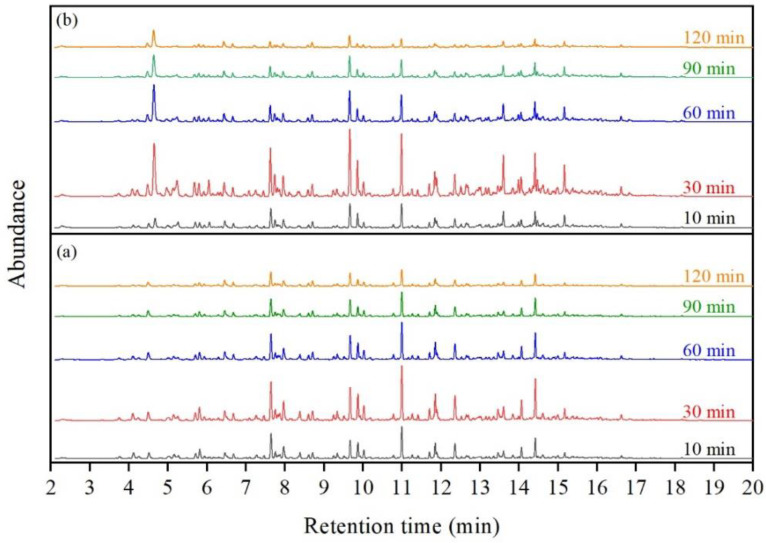
VOCs GC-MS chromatograms of (**a**) CRMB and (**b**) Mt-CRMB binders at different times.

Figure 9 shows the VOCs GC-MS chromatograms of the CRMB and Mt-CRMB binders at different retention times, following sampling Method 2 (see Figure 2). The characteristic peak abundance of the CRMB and Mt-CRMB VOCs changed with the extension of the heating time. At Time 2 (i.e., 30 min), the peak abundance reached the maximum. It meant that the CRMB and Mt-CRMB binders at 180 °C had the most VOCs emission after heating for half an hour.

**Figure 10:**
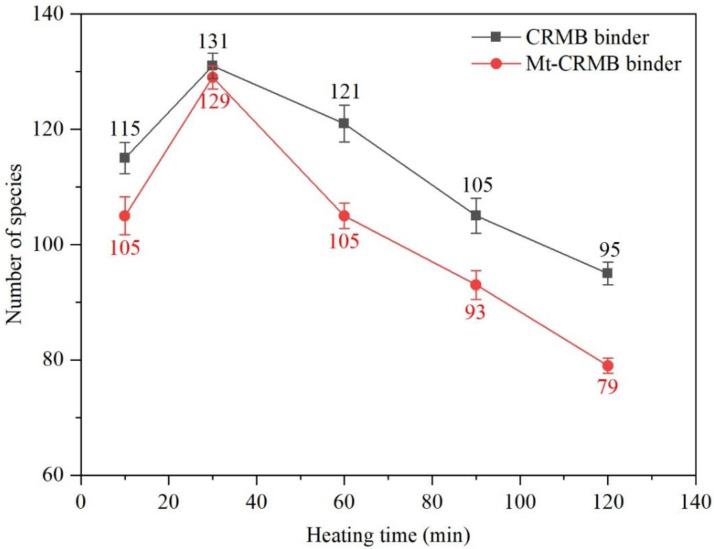
VOCs species number comparison between CRMB and Mt-CRMB binders at different times.

Figure 10 shows the species numbers of CRMB and Mt-CRMB VOCs as a function of the heating time. As indicated, the species number for both binders had a similar change trend. At the same time, the Mt-CRMB VOCs had a lower number. Both had a very close maximum number at 30 min. With the extension of the heating time, the species number gradually decreased. At 120 min (i.e., the fifth sampling), the Mt-CRMB binder only had 79 VOCs species, reduced by 39% compared with the maximum value. However, this number for the CRMB binder only decreased by 27%. It indicated that adding Mt could effectively reduce the number of the VOCs of the CRMB binder.

**Figure 11:**
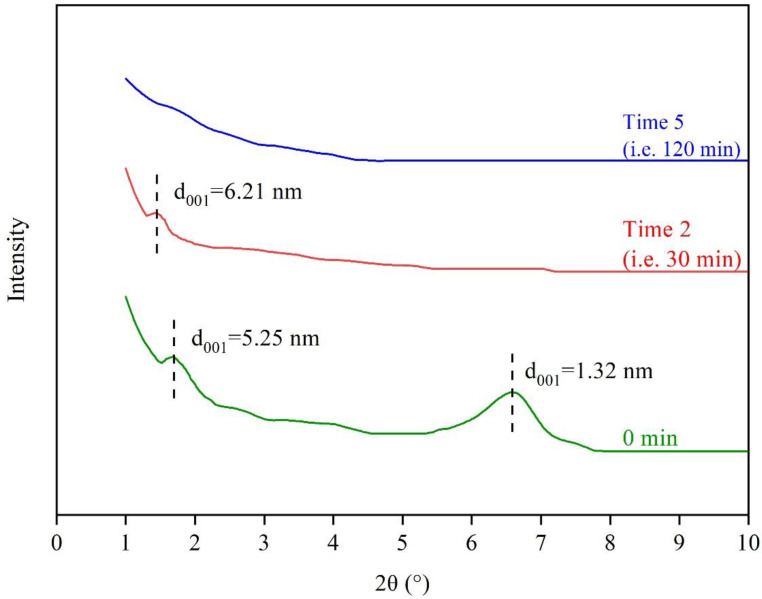
XRD spectrum of Mt-CRMB binder at different times.

Figure 11 shows the structural change of Mt in the CRMB binder at different times. For the time at 0 min, there were two diffraction peaks. According to the Bragg equation, the basal spacing of Mt was 1.32 nm at 6.62° and 5.25 nm at 1.68°. It meant that parts of Mt remained the original structure, but some showed an intercalated structure with a basal spacing increased by almost three times. The reason was that some small bituminous molecules entered the interlayer of the Mt, forming an intercalation structure [40]. The structure limited the release of bitumen VOCs and reduced their escape paths.

For the time at 30 min, there was only one diffraction peak at 1.42°. The peak for the original Mt at 6.62° disappeared, probably due to the continuous stirring of the magnetic rotor improving the exfoliated nanoclay. According to the Bragg equation, the basal spacing of Mt increased from 5.25 nm to 6.21 nm. For the time at 120 min, there was no prominent diffraction peak. The reason was that most Mt layers were exfoliated due to the stirring function. This exfoliation structure improved the inhibition effect on the VOCs emission, with the smallest species number at 120 min. 

### 3.3. VOCs Emission Model of Bitumen

#### 3.3.1. Establishment of Model

**Figure 12:**
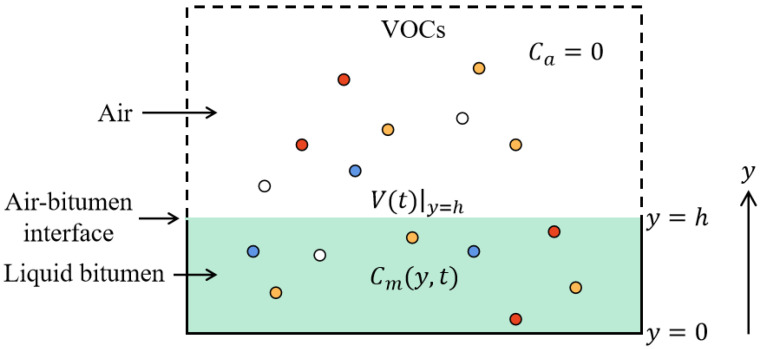
Schematic diagram of VOCs emission from bitumen into the air.

Figure 12 shows the schematic diagram of VOCs emission from bitumen. Generally, the VOCs emission consists of internal molecular diffusion in the material, partition on the air–material interface, and external convective mass transfer in the ambient air [41]. Five assumptions were established in the model establishment: the initial concentration of VOCs was uniformly distributed, there was no chemical reaction during the VOCs diffusion, the concentration gradient of VOCs was considered to be the only mass transfer force, the equilibrium condition existed between the material surface and the air boundary layer, and the partition on the material–air interface and the external convective mass transfer resistance at the air–material interface can be ignored. Under the above assumptions, the bitumen VOCs emission can be regarded as one-dimensional, and its emission rate measured in the experiment equaled that at the upper surface of bitumen.

The VOCs diffusion in bitumen follows Fick’s second law and can be described by the unsteady diffusion equation:

(3) $$\frac{\partial C_{m}}{\partial t}=\frac{\partial }{\partial y}\left(D_{m}\frac{\partial C_{m}}{\partial y}\right),\;0\leq y\leq h$$

where, $C_{m}$ is the concentration of each VOC in bitumen, μg/m^3^, $D_{m}$ is the diffusion coefficient of each VOC in bitumen, m^2^/s, and h is the thickness of the bitumen sample, m.

The initial condition of VOCs is given as:

(4) $${C_{m}|}_{t=0}=C_{0},\;0\leq y\leq h$$

where, $C_{0}$ is the initial concentration of each VOC inside the bitumen, μg/m^3^, and t is the heating time, s.

Since the bitumen samples will not penetrate the glass substrate, the concentration gradient is 0 μg/m^4^ (i.e., no mass flux) on the bottom boundary. It means that:

(5) $${\frac{\partial C_{m}}{\partial y}|}_{y=0}=0$$

Since the operation process shown in Figure 2 was designed to simulate the open environment in the process of bitumen preparation, transportation, and spreading in the actual situation, the spread of VOCs emitted from bitumen to the external environment is infinite. Therefore, the assumption of zero concentration on the upper surface of the sample is valid in this case, as follows:

(6) $${C_{m}|}_{y=h}=0$$

When the time, t, is long enough, the model for the instantaneous emission rate of VOCs based on the Laplace transform can be expressed as [41]:

(7) $$V\left(t\right)=\frac{2D_{m}C_{0}}{h}e^{\frac{-D_{m}\pi^{2}}{4h^{2}}t}$$

Equation (7) can also be expressed as:

(8) $$lnV\left(t\right)=ln\left(\frac{2D_{m}C_{0}}{h}\right)-\frac{D_{m}\pi^{2}}{4h^{2}}t$$

V(t) can be obtained from the experiment and calculated by the following Equation:

(9) $$V\left(t\right)=\frac{m_{e}}{St_{c}}$$

where,$\;m_{e}$ is the mass of each compound measured in the experiment, μg, S is the area of the upper surface of the sample, m^2^, and $\;t_{c}$ is the closed time before each sampling, min.

Two parameters, $C_{0}$ and $D_{m}$, can be obtained through the linear curve fitting based on Equation (8).

**Figure 13:**
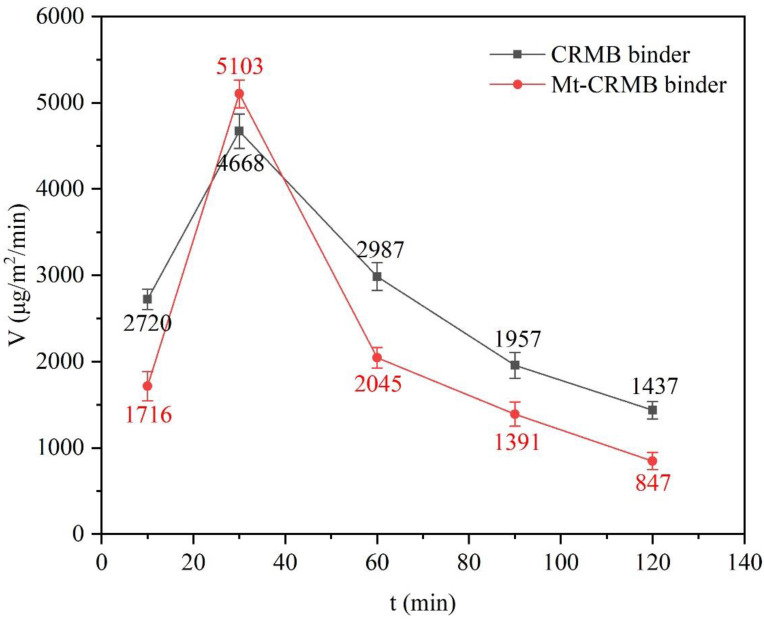
Total VOCs emission rates of CRMB and Mt-CRMB binders.

Figure 13 shows the total VOCs emission rates of CRMB and Mt-CRMB binders, and both total emission rates of the two binders sharply increased before 30 min and then gradually decreased. The VOCs emission rates of CRMB and Mt-CRMB binders were close at 30 min. However, with the extension of the heating time, their VOCs emission rates were significantly different. At 120 min, this value of the Mt-CRMB binder decreased by 41% compared with CRMB. For the VOCs collection device, a hysteresis of heat transfer existed at the initial state, leading to the uneven distribution of the VOCs concentration inside the binder. Therefore, there was an emission peak at 30 min. After this time, the internal concentration tended to be uniform, and the emission of bitumen VOCs met Fick’s law. Therefore, the emission rates at 30, 60, 90, and 120 min were used to fit the model of Equation (7).

Table 4 presents the VOCs emission rate of each fingerprint compound. In comparison, the emission rates for all fingerprint compounds of the Mt-CRMB binder decreased at the same heating time, except for No. 12 and No. 21 compounds. It indicated that adding Mt significantly reduced the emission rate of most VOCs in the bitumen.

Table 5 presents the initial concentration, $C_{0}$, and the diffusion coefficient, $D_{m}$, in the emission model for 23 fingerprint compounds of CRMB and Mt-CRMB binders. As indicated, $C_{0}$ for most compounds after the Mt modification decreased, except for undecane. The acetone had the highest $C_{0}$ of 2.033 × 10^6^ μg/m^3^, and this value decreased by 27% in the Mt-CRMB binder. Meanwhile, $C_{0}$ of 2-methyl-furan, 2-methyl-heptane, octane, and mesitylene decreased by 53%, 58%, 51%, and 51%, respectively. For most compounds, $D_{m}$ was relatively stable after the Mt modification.

#### 3.3.2. The Model Validation

**Figure 14:**
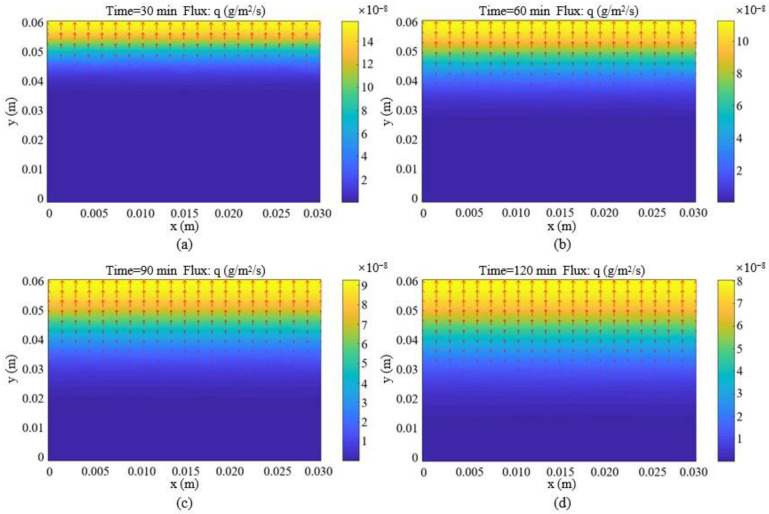
Two-dimensional cloud images of benzothiazole diffusion in CRMB binder at (**a**) 30 min, (**b**) 60 min, (**c**) 90 min, and (**d**) 120 min.

For validating the emission model of Equation (8), the Concentration Field of the PDEtoolbox in MATLAB software was used to construct the finite element model (FEM) for four typical components (toluene, styrene, benzothiazole, and methyl isobutyl ketone) in the CRMB and Mt-CRMB binders. In this FE model, a rectangle was selected as the model’s shape and gridded. The boundary (left, right, and bottom) conditions were set with the diffusion concentration gradient equal to 0 μg/m^4^. The parameters of $C_{0}$ and $D_{m}$ in Table 5 were input into the software to obtain the diffusion model of VOCs inside bitumen. Figure 14 shows the two-dimensional cloud image of benzothiazole diffusion in the CRMB binder at different times. At the same time, the diffusion flux from the bottom to the surface gradually increased. As the volatilization time increased, the diffusion flux at the upper interface gradually decreased.

**Figure 15:**
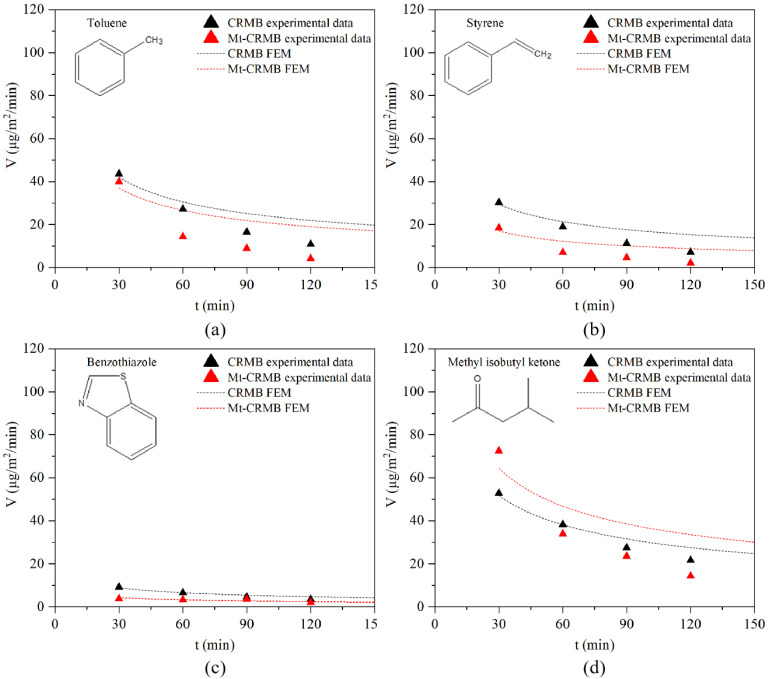
Emission rate of four typical compounds of (**a**) toluene, (**b**) styrene, (**c**) benzothiazole, and (**d**) methyl isobutyl ketone at the upper interface based on the FEM and the experiment.

Figure 15 shows the emission rate of four typical compounds at the upper interface based on the FEM and the experiment. As indicated, the emission rate gradually decreased with the increase in the heating time. The emission curve of these compounds in the CRMB and Mt-CRMB binders agreed well with the experimental data. In comparison, the CRMB binder better fit the model and the experiment than the Mt-CRMB. It was observed that the data at 90 min and 120 min differed more from the curve than at 30 min and 60 min. The reason could be that the bitumen gradually aged, hindering the VOCs emission. Although only four fingerprint compounds were used for the verification, it was still proven that the analytical solutions derived from Equations (3)–(6) were reasonable and can be used to describe the emission behavior of bitumen very well. This model can provide a concise and efficient understanding of bitumen VOCs emission behavior and lays a solid foundation for further deepening the model (considering more influencing factors) in future studies.

## 4. Conclusions

The VOCs emission characteristics of base and CRMB binders during heating were characterized and the inhibiting effect of Mt on the VOCs emission of CRMB was investigated in this research. A VOCs emission model of bitumen was established to compare the VOCs emission behaviors of CRMB and Mt-CRMB. Based on the results and discussion, the following conclusions can be drawn:

(1) The addition of crumb rubber increased the VOCs species number from 123 to 144. The VOCs emission of the CRMB binder was 3.2 times larger than that of the base binder. Furthermore, the addition of crumb rubber significantly increased the ARHs of the base binder, and the emission of some ARHs such as benzene, toluene, m-xylene, and p-xylene emissions increased by more than 10 times. However, the emission of some lightweight compounds such as alkanes was reduced since they were absorbed by the rubber.

(2) Due to its special intercalated structure, the VOCs of Mt-CRMB included 108 compounds, 25% less than that of CRMB, and the Mt nanoclay can reduce the VOCs emission of CRMB binder by about 30%. Especially, its inhibition effects on alkanes, olefins, and aromatic hydrocarbons were more significant, and 14 fingerprint compounds belonging to the above types were reduced by more than 30%. With the extension of the heating time, the VOCs species number of CRMB and Mt-CRMB binders gradually decreased. At 120 min, the Mt-CRMB binder only emitted 79 species of VOCs, reduced by 39% compared with the maximum value.

(3) The VOCs emission rates of CRMB and Mt-CRMB binders were close at 30 min. However, with the extension of the heating time, their VOCs emission rates were significantly different. The emission rate of the Mt-CRMB binder decreased by 41% compared with the CRMB binder at 120 min. Furthermore, the addition of Mt significantly reduced the emission rate of most VOCs in the bitumen. By calculation, the $C_{0}$ for most compounds after the Mt modification decreased, except for undecane, and for most compounds, the $D_{m}$ was relatively stable after the Mt modification. Based on Fick’s second law, the emission behavior of CRMB and Mt-CRMB binders can be well-described by the established model. 

Overall, the Mt nanoclay can be used as an effective modifier to inhibit the VOCs emission of the CRMB binder, which is widely used in asphalt pavement.

## Figures and Tables

**Table 1:** Basic properties of base bitumen.

Properties	Values	Standard
Penetration at 25 °C (0.1 mm)	66	ASTM D5
Softening point (°C)	48	ASTM D36
Ductility at 15 °C (cm)	181	ASTM D113
Viscosity at 60 °C (Pa·s)	166	ASTM D4402
Viscosity at 135 °C (Pa·s)	0.54	ASTM D4402

**Table 2:** Physical properties of montmorillonite.

Properties	Values
Basal spacing (nm)	1.3
Density (g/cm^3^)	1.7538
Appearance	White powder
Weight content of surfactant (%)	9

**Table 3:** The 23 VOCs fingerprint compounds from 3 areas and their standard curves for the GC-MS.

Area Code	No.	Retention Time (min)	Compounds	Standard Curve
1	1	3.743	Pentane	y = 6443x
	2	4.499	Acetone	y = 34,348x
	4	5.807	n-Hexane	y = 32,620x
	5	5.924	Methacrolein	y = 43,341x
	8	7.451	3-methyl-hexane	y = 57,274x
	9	7.632	Benzene	y = 116,197x
	10	7.958	Heptane	y = 82,427x
	11	9.326	2-methyl-heptane	y = 58,003x
	13	9.854	Toluene	y = 137,374x
	14	10.006	Octane	y = 160,012x
	16	11.680	m-Xylene	y = 74,470x
	17	11.825	p-Xylene	y = 137,036x
2	3	5.143	2-methyl-pentane	y = 41,944x
	7	6.453	Butanal	y = 33,394x
	19	14.016	Mesitylene	y = 138,233x
	21	15.115	Undecane	y = 124,317x
	22	17.369	Naphthalene	y = 466,879x
3	6	6.288	2-methyl-furan	y = 58,003x
	12	9.656	Methyl Isobutyl Ketone	y = 146,885x
	15	10.974	4-ethenyl-cyclohexene	y = 70,311x
	18	12.331	Styrene	y = 92,293x
	20	14.367	D-Limonene	y = 121,411x
	23	18.080	Benzothiazole	y = 24,376x

**Table 4:** Emission rate, V(t), of 23 fingerprint compounds for CRMB and Mt-CRMB binders at different times.

No.	Emission Rate (μg/m^2^/min)									
	Sampling Time for CRMB Binder					Sampling Time for Mt-CRMB Binder				
	1	2	3	4	5	1	2	3	4	5
1	117.00	160.73	99.80	63.50	45.32	46.52	111.93	44.42	35.73	33.12
2	92.65	142.55	129.52	111.15	72.33	53.28	139.05	88.20	75.12	53.47
3	33.50	44.68	27.95	17.85	12.03	13.85	43.35	15.48	8.27	5.10
4	54.98	79.20	45.92	29.37	21.05	20.52	52.52	21.47	15.63	11.23
5	12.22	18.32	15.58	12.38	10.80	8.72	16.17	10.40	8.68	7.23
6	4.78	9.32	8.23	6.72	5.93	3.08	8.73	4.68	4.05	2.95
7	26.68	46.28	38.48	29.18	27.80	21.62	44.07	26.00	20.27	19.37
8	8.75	14.13	8.77	5.78	4.12	3.85	11.00	4.37	3.02	1.70
9	64.10	98.18	64.57	45.12	35.60	34.90	83.05	28.52	19.17	10.42
10	28.97	39.13	23.53	15.65	12.27	13.60	28.93	12.50	9.17	6.93
11	13.32	23.43	14.48	9.28	6.30	4.52	13.05	5.32	3.40	1.88
12	29.72	52.68	38.22	27.48	21.58	26.30	72.45	33.83	23.50	14.33
13	26.67	43.55	27.17	16.47	10.82	16.42	39.88	15.24	8.85	4.08
14	7.73	13.32	8.43	5.48	3.93	3.28	8.88	3.92	2.72	1.83
15	48.85	83.82	56.92	37.22	25.50	24.37	64.00	27.27	17.98	8.83
16	22.37	37.70	24.22	14.97	9.92	10.92	26.78	10.33	6.83	3.35
17	21.93	37.07	24.28	15.47	10.50	9.48	22.83	9.25	6.32	3.27
18	18.38	30.13	18.88	11.17	7.05	8.00	18.42	6.93	4.47	2.08
19	13.30	23.73	15.60	10.08	6.88	6.08	13.60	6.32	4.52	2.52
20	14.37	29.52	18.43	12.68	8.70	7.38	19.47	9.02	6.47	3.52
21	5.28	10.48	6.65	4.23	2.97	7.60	18.62	8.65	6.03	3.18
22	0.37	0.67	0.48	0.30	0.22	0.25	0.50	0.30	0.23	0.15
23	5.77	9.18	6.60	4.50	3.47	2.07	3.65	3.28	3.53	2.07
Total SD	121.53	198.75	162.88	155.47	99.21	171.54	160.15	122.34	142.78	104.12

**Table 5:** The initial concentration, $C_{0}$, and the diffusion coefficient, $D_{m}$, in the emission model for 23 fingerprint compounds of CRMB and Mt-CRMB binders.

No.	CRMB Binder			Mt-CRMB Binder		
	C_0_ (μg/m^3^)	D_m_ (m^2^/s)	R^2^	C_0_ (μg/m^3^)	D_m_ (m^2^/s)	R^2^
1	1.355 × 10^6^	2.163 × 10^−8^	0.9914	1.001 × 10^6^	7.439 × 10^−9^	0.6893
2	2.033 × 10^6^	1.607 × 10^−8^	0.8316	1.479 × 10^6^	1.086 × 10^−8^	0.9538
3	3.780 × 10^5^	3.182 × 10^−8^	0.9978	2.135 × 10^5^	2.229 × 10^−8^	0.9540
4	6.198 × 10^5^	2.266 × 10^−8^	0.9829	3.144 × 10^5^	1.673 × 10^−8^	0.8896
5	3.058 × 10^5^	1.302 × 10^−8^	0.9865	1.801 × 10^5^	8.930 × 10^−9^	0.9151
6	1.734 × 10^5^	1.592 × 10^−8^	0.9849	8.160 × 10^4^	7.839 × 10^−9^	0.8928
7	7.445 × 10^5^	1.247 × 10^−8^	0.9155	4.265 × 10^5^	9.344 × 10^−9^	0.7946
8	1.198 × 10^5^	2.919 × 10^−8^	0.9917	7.308 × 10^4^	2.089 × 10^−8^	0.9513
9	9.257 × 10^5^	2.718 × 10^−8^	0.9784	4.840 × 10^5^	3.094 × 10^−8^	0.9399
10	3.250 × 10^5^	1.974 × 10^−8^	0.9657	1.888 × 10^5^	1.490 × 10^−8^	0.8808
11	1.959 × 10^5^	2.978 × 10^−8^	0.9966	8.314 × 10^4^	2.230 × 10^−8^	0.9693
12	5.721 × 10^5^	2.509 × 10^−8^	0.9932	5.173 × 10^5^	1.524 × 10^−8^	0.9660
13	3.651 × 10^5^	3.385 × 10^−8^	0.9983	2.769 × 10^5^	3.849 × 10^−8^	0.9711
14	1.144 × 10^5^	2.194 × 10^−8^	0.9925	5.576 × 10^4^	2.024 × 10^−8^	0.9378
15	7.753 × 10^5^	3.009 × 10^−8^	0.9993	4.629 × 10^5^	3.344 × 10^−8^	0.9767
16	3.260 × 10^5^	3.513 × 10^−8^	0.9988	1.906 × 10^5^	2.255 × 10^−8^	0.9672
17	3.305 × 10^5^	3.288 × 10^−8^	0.9988	1.661 × 10^5^	2.125 × 10^−8^	0.9648
18	2.492 × 10^5^	3.679 × 10^−8^	0.9991	1.293 × 10^5^	2.453 × 10^−8^	0.9708
19	2.125 × 10^5^	2.848 × 10^−8^	0.9990	1.047 × 10^5^	2.114 × 10^−8^	0.9680
20	2.579 × 10^5^	2.888 × 10^−8^	0.9950	1.489 × 10^5^	1.946 × 10^−8^	0.9687
21	9.030 × 10^4^	2.148 × 10^−8^	0.9949	1.406 × 10^5^	2.981 × 10^−8^	0.9746
22	6.363 × 10^3^	2.087 × 10^−8^	0.9948	4.483 × 10^3^	1.874 × 10^−8^	0.9700
23	9.552 × 10^4^	1.669 × 10^−8^	0.9929	5.750 × 10^4^	9.606 × 10^−9^	0.4464

## Data Availability

Data are contained within the article.
